# Interleukin-1 Stimulates ADAM17 through a Mechanism Independent of its Cytoplasmic Domain or Phosphorylation at Threonine 735

**DOI:** 10.1371/journal.pone.0031600

**Published:** 2012-02-27

**Authors:** Katherine C. Hall, Carl P. Blobel

**Affiliations:** 1 Arthritis and Tissue Degeneration Program, The Hospital for Special Surgery, New York, New York, United States of America; 2 Cell Biology and Genetics Program, Weill Medical College of Cornell University, New York, New York, United States of America; 3 Department of Medicine and Physiology, Biophysics and Systems Biology Program, Weill Medical College of Cornell University, New York, New York, United States of America; The University of Queensland, Australia

## Abstract

ADAM17 (a disintegrin and metalloproteinase) is a membrane-anchored metalloproteinase that regulates the release of EGFR-ligands, TNFα and other membrane proteins from cells. ADAM17 can be rapidly activated by a variety of signaling pathways, yet little is known about the underlying mechanism. Several studies have demonstrated that the cytoplasmic domain of ADAM17 is not required for its rapid activation by a variety of stimuli, including phorbol esters, tyrosine kinases and some G-protein coupled receptors. However, phosphorylation of cytoplasmic residue T735 was recently reported as a crucial step for activation of ADAM17 by IL-1β and by the p38 MAP-kinase pathway. One possible mechanism to reconcile these results would be that T735 has an inhibitory role and that it must be phosphorylated as a pre-requisite for the activation of ADAM17, which would then proceed via a mechanism that is independent of its cytoplasmic domain. To test this hypothesis, we performed rescue experiments of *Adam17−/−* cells with wild type and mutant forms of ADAM17. However, these experiments showed that an inactivating mutation (T735A) or an activating mutation (T735D) of cytoplasmic residue T735 or the removal of the cytoplasmic domain of ADAM17 did not significantly affect the stimulation of ADAM17 by IL-1β or by activation of MAP-kinase with anisomycin. Moreover, we found that the MAP-kinase inhibitor SB203580 blocked activation of cytoplasmic tail-deficient ADAM17 and of the T735A mutant by IL-1β or by anisomycin, providing further support for a model in which the activation mechanism of ADAM17 does not rely on its cytoplasmic domain or phosphorylation of T735.

## Introduction

ADAM17 (a disintegrin and metalloproteinase 17) is a membrane-anchored metalloproteinase that has crucial roles in regulating the release of the pro-inflammatory cytokine tumor necrosis factor α (TNFα) [1]–[4] as well as the bioavailability of ligands of the epidermal growth factor receptor (EGFR) [5]–[9], and has been implicated in the ectodomain shedding of numerous other membrane proteins (reviewed in [5], [10]). ADAM17 is important for proper development of the skin, lung, mammary gland and heart valves [6]–[9], and it has emerged as a critical mediator of TNF-dependent endotoxin shock, pathological neovascularization and of intestinal inflammation and regeneration in adult mice [2], [11], [12]. The sheddase activity of ADAM17 can be rapidly activated in response to a variety of different stimuli in cell-based assays [8], [13]–[20], yet much remains to be learned about the underlying mechanism. Reddy et al. [13] reported that phorbol ester-stimulated shedding of TNFα and other membrane proteins from *Adam17−/−* mouse embryonic fibroblasts (mEFs) could be restored by a mutant form of ADAM17 lacking its cytoplasmic domain, including all cytoplasmic phosphorylation sites, thereby demonstrating that the activation of ADAM17 by phorbol 12-myristate 13-acetate (PMA) does not require cytoplasmic phosphorylation. Later, a similar approach was used to show that the response of ADAM17 to treatment of cells with other stimuli, including physiological stimuli such as epidermal growth factor (EGF), TNFα, lysophosphatidic acid, thrombin, benzoyl-ATP and fibroblast growth factor 7 as well as oncogenic Src, the calcium ionophore Ionomycin, or the mercurial compound acetyloxy-(4-aminophenyl)mercury [15], [16], [21] required the transmembrane domain, but not the cytoplasmic domain of ADAM17.

Even though the cytoplasmic domain of ADAM17 is not necessary for its ability to respond to the stimuli listed above, several studies have demonstrated that this domain is phosphorylated when cells are treated with stimuli such as PMA, EGF, nerve growth factor [22], lipopolysaccharide [23], fibroblast growth factor, fetal bovine serum (FBS) [24], the GPCR agonist gastrin-related peptide [25], transforming growth factor β [26] and interleukin-1β (IL-1β) [27]. Moreover, Diaz-Rodriguez et al. [22] showed that a threonine to alanine mutation at cytoplasmic residue 735 (T735>A) in ADAM17 resulted in 41% less processing of neurotrophic tyrosine kinase receptor A compared to wild type ADAM17 following treatment with PMA. More recently, Xu and Derynck reported that the T735>A mutation almost completely abrogated the ability of ADAM17 to respond to stimulation by IL-1β or anisomycin, which activate the p38 MAPK pathway [27]. Additionally, Xu and Derynck provided evidence for a direct interaction between p38 and the ADAM17 cytoplasmic domain, and therefore proposed that phosphorylation of T735 by p38 MAPK is a key event in the activation of ADAM17 in response to IL-1β.

The finding that ADAM17 requires cytoplasmic phosphorylation of T735 in order to respond to activation by IL-1β, but not by PMA [27], and the observation that the cytoplasmic domain is dispensable for activation of ADAM17 by PMA [13], EGF, TNFα, lysophosphatidic acid, thrombin, Ionomycin, benzoyl-ATP [15], oncogenic Src [21] and activation of fibroblast growth factor receptor 2b [16] raised questions about the cause for these apparent discrepancies. One possibility was that ADAM17 relies on distinct mechanisms to respond to different stimuli, such that the cytoplasmic domain is dispensable for activation by the stimuli listed above, but not for stimulation by IL-1β or anisomycin. Another possible explanation was that the cytoplasmic residue T735 functions to prevent the activation of ADAM17, and that this residue must be phosphorylated as a pre-requisite for the activation of ADAM17. In this scenario, the T735>A mutation would block the ability of ADAM17 to respond to stimulation, whereas removal of the cytoplasmic domain would relieve any inhibitory effect of T735 on ADAM17, thus allowing it to respond to various stimuli. The main goal of the present study was to further define the role of T735 and of the cytoplasmic domain of ADAM17 in its regulation by IL-1β and anisomycin.

## Materials and Methods

### Ethics Statement

The isolation of primary mEFs was approved by the Internal Animal Care and Use Committee of the Hospital for Special Surgery (protocol # 09-10-22M, approval date 10/27/2010).

### Reagents

All reagents were from Sigma unless indicated otherwise. Bisindolylmaleimide I (BIM I), SB203580, and anisomycin were from Calbiochem, and recombinant mouse IL-1β was purchased from R&D Systems or Peprotech. Both IL-1β preparations were tested for their ability to activate ADAM17 in parallel and were found to cause equivalent activation. Marimastat was kindly provided by Dr. Ouathek Ouerfelli (Sloan-Kettering Institute, New York, NY).

### Expression constructs

Alkaline phosphatase (AP)-tagged transforming growth factor α (TGFα), betacellulin (BTC), CD62L, and a TNFα cleavage site construct as well as C-terminally hemagglutinin (HA)-tagged murine ADAM17 and ADAM17ΔC lacking its cytoplasmic domain have been previously described [15], [28], [29]. The cDNA for human TNFα was cloned from the pFLAG-CMV2 [30] into the pCDNA3.1(-) A myc/his vector. The cDNA for wild type (wt) HA-tagged murine ADAM17 was used to generate a catalytically inactive Glu406 to Ala406 mutant (ADAM17E>A-HA) as well as the cytoplasmic Thr735 to Ala735 mutant (T735A) to abolish this phosphorylation site, or the Thr735 to Asp735 mutant (T735D) to mimic the phosphorylated threonine. Separate PCR reactions were performed to create untagged T735A and T735D mutants of ADAM17. The cDNA for human interleukin-1 receptor 2 (IL-1R2) was cloned into the pAPtag5 vector to create an IL-1R2-AP fusion protein (Residue 14 to 398). Successful introduction of the desired mutations and the absence of other mutations were confirmed by sequencing. Constructs expressing human wild type ADAM17 and the human ADAM17-T735A mutant were generously provided by Drs. P. Xu and R. Derynck (Univ. of California, San Francisco).

### Cell lines and culture conditions

**Figure 1:**
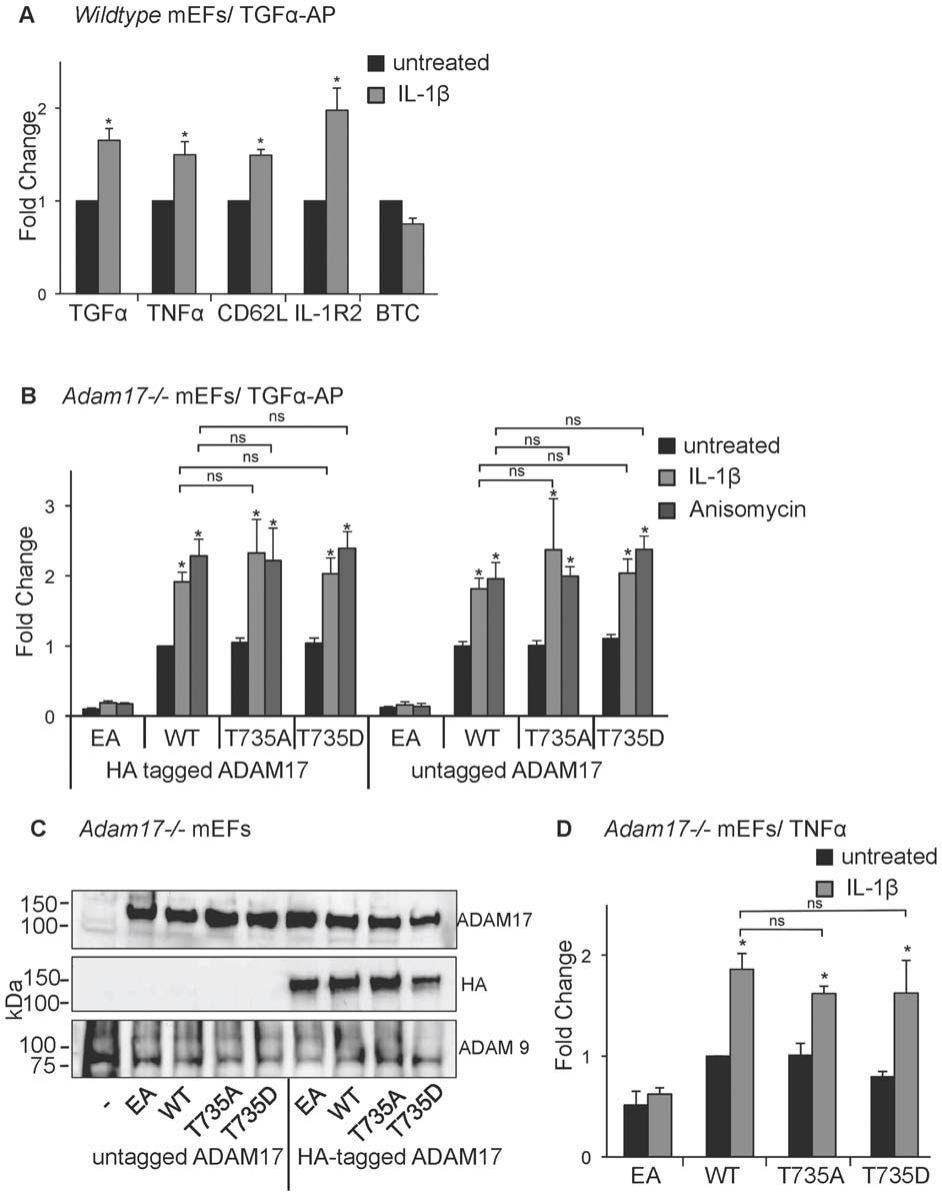
Stimulation of wild type and T735 mutant forms of ADAM17 by IL-1ß and anisomycin. A) Wild type mEFs were transfected with alkaline phosphatase-tagged substrates to monitor the sheddase activity of ADAM17 (TGFα, TNFα, CD62L and IL-1R2) or ADAM10 (BTC) and stimulated with IL-1ß (5 ng/ml) for 30 minutes. The release of the AP-tagged ADAM10 or ADAM17 substrates into the culture supernatant was determined as described in materials and methods. B) *Adam17−/−* primary mEFs were transfected with TGFα-AP and either HA-tagged or untagged forms of the catalytically inactive ADAM17-E406A (EA), wild type ADAM17 (WT), or ADAM17 carrying point mutations at residue T735 (T735A or T735D), as indicated. The amount of TGFα-AP released in 30 minutes from untreated cells (black bars) or from cells stimulated with IL-1ß (5 ng/ml, light grey bars) or anisomycin (1 µM, dark grey bars) was determined as described in materials and methods. C) Western blots of untagged or HA-tagged forms of ADAM17 (EA, WT, T735A or T735D) expressed in *Adam17−/−* mEFs were probed with antibodies against the cytoplasmic domain of ADAM17 (top panel) or the HA-tag (middle panel), or against ADAM9 as a loading control. D) Shedding of transfected human TNFα from *Adam17−/−* mEFs rescued with WT or the T735A, T735D or EA mutant forms of ADAM17. The amount of TNFα released into the conditioned media from unstimulated cells or cells stimulated with IL-1β (5 ng/ml) in 30 minutes was determined by ELISA. All data represent the average of at least three separate experiments, and the error bars correspond to +/− standard error of the mean (sem). Asterisks indicate a significant increase upon addition of the stimulatory agent (students t-test, p<0.05). NS indicates that no significant difference was found between the indicated conditions.

**Figure 2:**
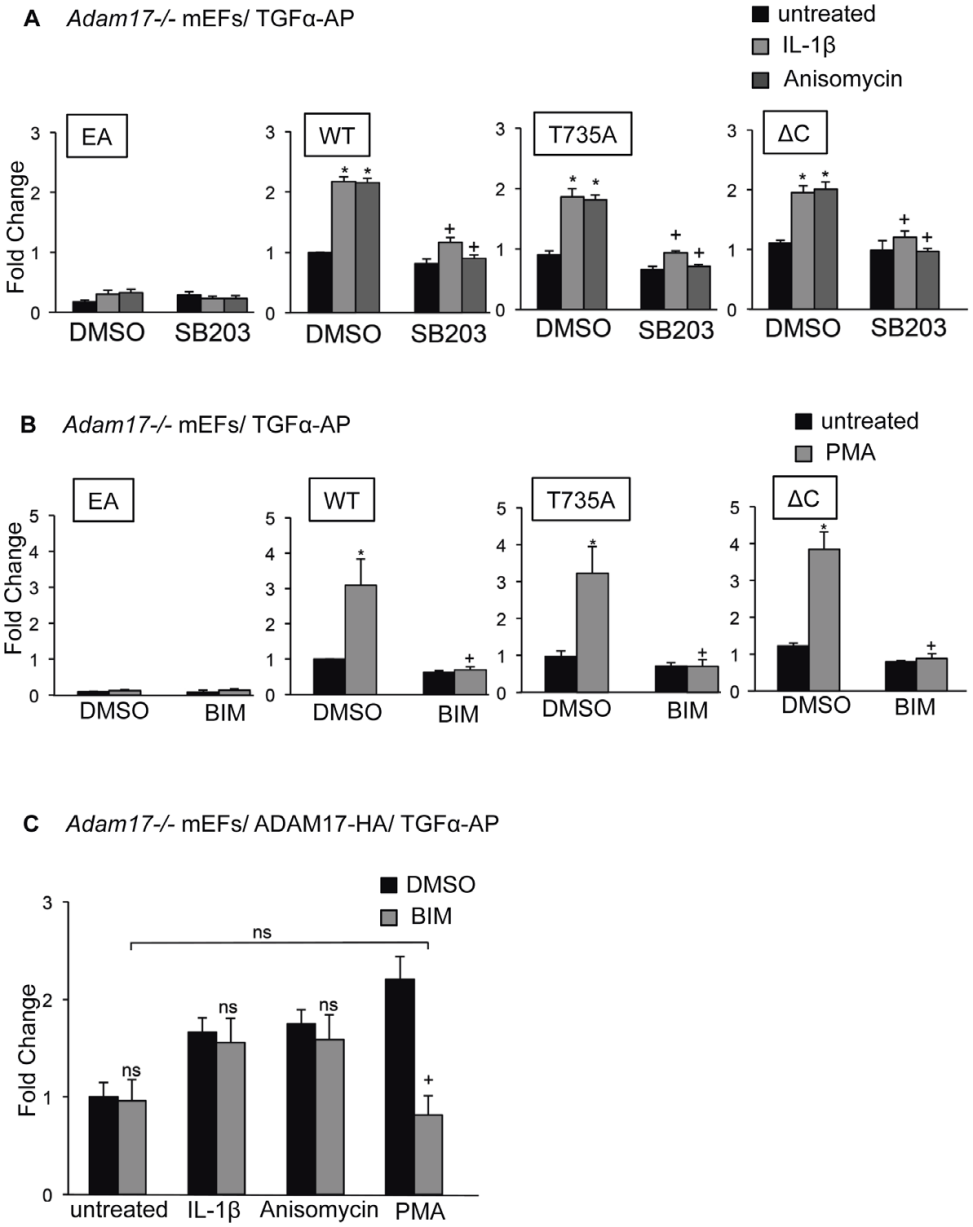
Effect of inhibitors of p38 MAPK or PKC on stimulated shedding by ADAM17T735A and ADAM17ΔC. A) *Adam17−/−* mEFs were co-transfected with TGFα-AP and murine WT ADAM17, EA, T735A or ΔC mutants, and either not treated (black bars) or stimulated with IL-1β (5 ng/ml, light grey bars) or anisomycin (1 µM, dark grey bars), in the presence or absence of 10 µM of the p38 MAPK inhibitor SB203580 (SB203) as indicated, with DMSO serving as a control. Constitutive and stimulated shedding of TGFα-AP was rescued by cotransfection with WT ADAM17 as well as the T735A and ΔC mutants, but not by the inactive EA mutant. There was no significant difference between the constitutive or stimulated shedding of TGFα-AP from *Adam17−/−* mEFs rescued with WT compared to the T735A or ΔC mutant of ADAM17. IL-1β and anisomycin-stimulated shedding of TGFα-AP in cells rescued with WT, T735 or ΔC was blocked to a comparable extent by 10 µM of the p38 MAPK inhibitor SB203580. B) TGFα-AP shedding from *Adam17−/−* cells rescued with WT ADAM17, T735A or ΔC was stimulated to a comparable level by 25 ng/ml PMA (untreated, black bars, PMA-treated, grey bars), and the stimulation could be blocked by the PKC inhibitor BIM I (4 µM). C) *Adam17−/−* mEFs transfected with TGFα together with WT ADAM17 were stimulated with 5 ng/ml IL-1β, 1 µM anisomycin or 25 ng/ml PMA in the presence or absence of 4 µM BIM I. BIM I blocked the TGFα-AP shedding stimulated by PMA, but had no significant effect on the shedding stimulated by IL-1β or anisomycin. All results represent the average values of at least 3 experiments, and the error bars indicate +/− standard error of the mean (sem). Asterisks indicate a significant increase upon addition of a stimulus, whereas plus (+) indicates a significant decrease in stimulated shedding upon introduction of an inhibitor (students t-test, p<0.05).

**Figure 3:**
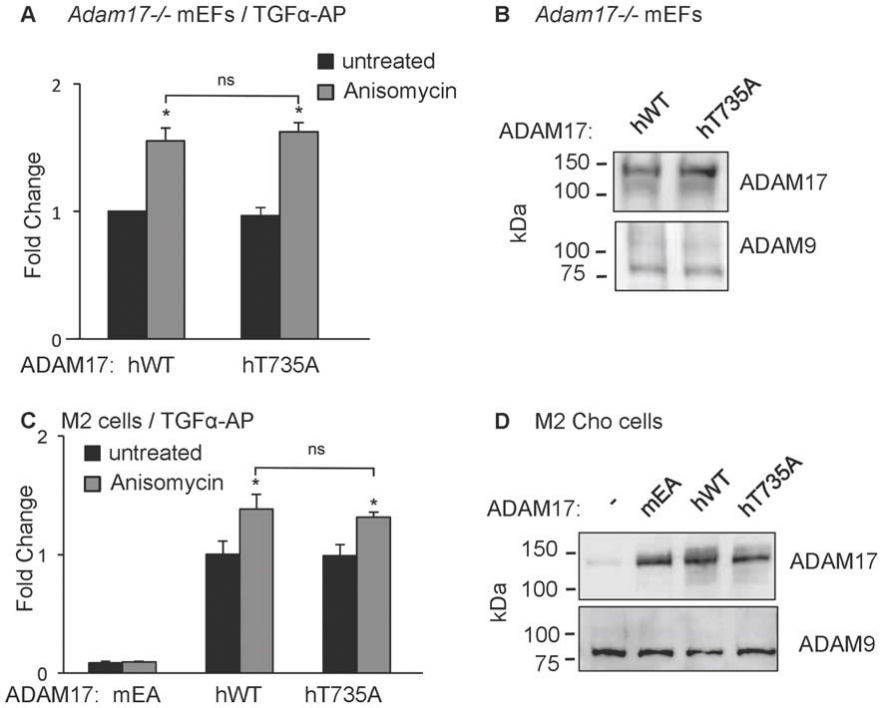
Comparison of TGFα shedding by human wild type ADAM17 and the T735A mutant. A) *Adam17−/−* mEFs were transfected with TGFα-AP together with human wild type ADAM17 (hWT) or the human ADAM17-T735A mutant (hT735A). TGFα-AP shedding was measured in supernatants conditioned for 30 minutes on untreated cells (black bars) or cells treated with 1 µM anisomycin (grey bars). There was no significant difference between constitutive or anisomycin-stimulated shedding by hT735A compared to hWT ADAM17. B) Western blot analysis with antibodies against the cytoplasmic domain of ADAM17 demonstrated comparable expression of hWT and hT735A, with ADAM9 serving as a loading control. C) M2 CHO cells carrying inactivating mutations in both alleles of ADAM17 [37] were transfected with TGFα-AP and the inactive mouse ADAM17EA to provide a negative control, or hWT or hT735A. Basal shedding of TGFα-AP was restored to comparable levels by the introduction of either hWT or hT735A. The stimulation of hWT and hT735 by 1 µM anisomycin was weaker in M2 CHO cells than in *Adam17−/−* mEFs rescued with these constructs, but there was no significant difference between the anisomycin-stimulated shedding by these two constructs. D) Western blot analysis of M2 CHO cells with anti-ADAM17 cytotail antibodies shows the expression of the endogenous mutant forms of ADAM17 in untransfected cells, and similar expression levels of murine ADAM17EA (mEA) compared to human WT or T735A ADAM17. The results in panels A and C represent the average of at least 3 experiments +/− sem. Asterisks indicate significant increase upon addition of a stimulus.

Wild type mEFs immortalized with large T-antigen (Figure 1A) have been previously described [2]. Primary *Adam17−/−* mEFs (Figures 1B–D, 2A–C and 3A,B) were isolated as described [8], [31] and genotyped by Western blotting with polyclonal antibodies against ADAM17 [32]. The ADAM17-deficient M2 CHO cell line [33], [34] (Figure 3C,D) was obtained from Dr. J. Arribas (Vall d'Hebron Institute of Oncology, Barcelona, Spain). All cells were grown in Dulbecco's modified Eagle's medium supplemented with 10% FBS and antibiotics, and were transfected using GenJet (SignaGen Laboratories) for 5 hours, and then transferred to growth medium for overnight recovery. All shedding experiments were performed one or two days after transfection.

### Shedding Assays

Prior to ectodomain shedding experiments, the transfected cells were serum-starved in low-serum OptiMem (Gibco) for two hours (mEFs) or 24 hours (M2 CHO cells). For experiments with inhibitors, the cells were pre-incubated with the inhibitor for 10 minutes before the addition of the stimulating agent for an additional 30 minutes. SB203580 was used at 10 µM, BIM I at 4 µM, IL-1β at 5 ng/ml, PMA at 25 ng/ml and anisomycin at 1 µM. The shedding of AP-tagged proteins was measured as previously described [8], [31]. In brief, after stimulation, conditioned media was collected and cells were lysed in 2.5% TritonX-100, 1 mM EDTA, 1 mM 1,10 phenanthroline. AP activity was determined by incubating the conditioned media and lysates with p-nitrophenyl phosphate (Thermo Scientific) at 37°C followed by measuring the OD_405_. Shed TNFα was detected using the optEIA Human TNFα ELISA kit (BD Biosciences) according to the manufacturer's instructions.

### Western Blotting

For Western blot analysis, transfected cells were lysed in 1% TritonX-100 in PBS with 10 mM 1,10 phenanthroline, 5 mM marimastat and 1× protein inhibitor cocktail [35]. Glycoproteins were enriched using Concanavalin A-agarose lectin beads (GE Healthcare) and then separated on 10% SDS-polyacrylamide gels. Proteins were transferred to nitrocellulose membranes (Pall Life Sciences) and probed with anti-HA antibodies (Covance), or antibodies against the cytoplasmic domain of ADAM17 [32] followed by HRP-coupled anti-mouse-IgG or anti-rabbit-IgG (Promega) and detected using enhanced chemoluminescence (ECL, Amersham).

### Statistical Analysis

All experiments were conducted at least in triplicate and the results were averaged. Standard error of the mean was calculated using Microsoft Excel. A paired Student's t-test was used to compare the results generated from experiments with identically transfected cells with or without stimulation and inhibition. When the comparison was between identically treated cells transfected with different constructs; an unpaired t-test was used. A p-value of <0.05 was considered statistically significant.

## Results and Discussion

In order to determine whether IL-1β stimulates ADAM10 or ADAM17 in wild type mEFs, we measured the release of the alkaline phosphatase-tagged ADAM17 substrates TGFα [7], [8], TNFα [1]–[3], CD62L [36], IL-1R2 [13], and of the ADAM10 substrate betacellulin [8] from these cells following incubation with 5 ng/ml IL-1β for 30 minutes. Treatment with IL-1β stimulated the shedding of the ADAM17 substrates, but not of the ADAM10 substrate, demonstrating that under these conditions IL-1β activates ADAM17, but not ADAM10 (Figure 1A).

To corroborate that ADAM17 is required for the IL-1β-stimulated shedding of TGFα in mEFs, primary *Adam17−/−* mEFs were transfected with TGFα-AP together with wild type or mutant forms of ADAM17. No increase in TGFα-AP shedding was observed upon stimulation with IL-1β when the catalytically inactive HA-tagged ADAM17-E>A mutant was co-transfected. However, co-transfection of HA-tagged ADAM17 was sufficient to increase constitutive shedding and restore the stimulated shedding of TGFα in response to treatment with IL-1β or anisomycin (Figure 1B).

The ability to rescue IL-1β-stimulated shedding of TGFα-AP in *Adam17−/−* cells by co-transfection of HA-tagged ADAM17 allowed us to test how mutations of T735 affected the response of ADAM17 to stimulation with IL-1β or anisomycin. We found that the ADAM17-T735>A (T735A) as well as the ADAM17-T735>D (T735D) phosphomimetic mutant were able to rescue constitutive and IL-1β- or anisomycin-stimulated shedding of TGFα in *Adam17−/−* cells (Figure 1B). When we performed identical rescue experiments with untagged versions of wt ADAM17, T735A, T735D and of the inactive E>A mutant, the results were comparable to those obtained with the corresponding HA-tagged forms of ADAM17, arguing against the possibility that the HA-tag interferes with regulation of ADAM17 by T735 (Figure 1B). Western blot analysis with antibodies against the HA tag or the cytoplasmic domain of ADAM17 corroborated similar expression of the wild type and mutant forms of HA-tagged or untagged ADAM17 (Figure 1C). Overexpressed ADAM17 was mainly detected as a pro-form on Western blots, which is consistent with previous studies [14]. Nevertheless, the relatively small amounts of mature wild type or mutant forms of ADAM17 produced under these conditions are evidently sufficient to rescue the shedding defect in *Adam17−/−* mEFs. Finally, we tested the shedding of a non-AP tagged ADAM17 substrate, TNFα, using an ELISA assay. The constitutive and IL-1β-stimulated shedding of TNFα by the T735 mutants was not significantly different from that mediated by wt ADAM17 (Figure 1D).

These results suggested that IL-1β activates ADAM17 in a manner that does not rely on phosphorylation of T735. To provide additional evidence for this finding, we tested whether the p38 MAPK inhibitor, SB203580 (10 µM), which blocks IL-1β-dependent stimulation of ADAM17 [27], also affected the stimulation of the T735A mutant by IL-1β or anisomycin. As shown in Figure 2A, SB203580 prevented IL-1β- and anisomycin-stimulated activation of T735A as effectively as of wt ADAM17. Similar results were obtained with ADAM17ΔC (ΔC), which lacks the cytoplasmic domain, including all potential phosphorylation sites (Figure 2A). These results suggest that stimulation of ADAM17 by IL-1β or anisomycin does not require an interaction between p38 MAPK and the cytoplasmic domain of ADAM17 or the presence of T735 or other cytoplasmic phosphorylation sites. Moreover, we found that the protein kinase C (PKC) inhibitor, BIM I, blocked PMA-stimulated activation of T735A or ΔC to a similar extent as it blocked activation of wt ADAM17 (Figure 2B), suggesting that a direct interaction of PKC with the cytoplasmic domain of ADAM17 is also not necessary for the stimulation of ADAM17 by PMA [13], [14], [27]. Additionally, the role of PKC in p38 MAPK stimulation of ADAM17 was tested by adding BIM I to TGFα-AP expressing *Adam17−/−* mEFs rescued with ADAM17 and treated with IL-1β, anisomycin or PMA (Figure 2C). BIM I prevented the PMA-stimulated shedding of TGFα-AP, but had no significant effect on IL-1β- or anisomycin-dependent stimulation of ADAM17. These results suggest that PKC is not required for the activation of ADAM17 by p38 MAPK.

To address the possibility that human and mouse ADAM17 differ in their response to phosphorylation of T735, we performed rescue experiments with wt human ADAM17 or human ADAM17-T735A in primary *Adam17−/−* mEFs or in ADAM17-deficient M2 CHO cells [33], [34], [37], which were also used by Xu and Derynck [27]. We found no significant difference in the response of human wt ADAM17 and ADAM17-T735A to stimulation with anisomycin in *Adam17−/−* mEFs (Figure 3A) or in M2 CHO cells, where inactive murine ADAM17EA served as a negative control (Figure 3C). Western blot analysis confirmed comparable expression of human wt and T735 ADAM17 in *Adam17−/−* mEFs (Figure 3B) and in M2 CHO cells (Figure 3D).

These results argue against a model in which cytoplasmic phosphorylation is a crucial component of the mechanism of activation of ADAM17 by IL-1β or anisomycin. Instead, they support previous studies in which the cytoplasmic domain of ADAM17 was not required for its response to several other stimuli [14], [15], [36], [38]. Moreover, they argue against a model in which T735 is part of an inhibitory mechanism that keeps ADAM17 inactive and that must be phosphorylated as a prerequisite to allow activation of ADAM17. The outcome of the rescue experiments were also comparable when shedding of alkaline phosphatase-tagged TGFα or non-AP tagged TNFα were used as a readout for the activity of ADAM17, both of which are cleaved almost exclusively by ADAM17 under the conditions used in our assay [2], [14]. The lack of IL-1β- and anisomycin-stimulated shedding of TGFα or TNFα from *Adam17−/−* mEFs rescued with the inactive ADAM17-E>A demonstrates that no other enzyme can compensate for the loss of ADAM17 when mEFs are activated with these stimuli, as previously also shown for several other stimuli [14], [15], [36], [38]. Finally, essentially identical results were obtained in rescue experiments with two other ADAM17-deficient cell types, M2 CHO cells [33], [34], [37] and *Adam17−/−* primary mouse chondrocytes (see Figure S1). Thus, the cause for the discrepancy between the results presented here and those reported by Xu and Derynck remains to be established, but could be due to different experimental conditions. Recently, Li et al. described a mutant form of ADAM17 carrying a Cys to Tyr mutation in residue 600 in the cysteine-rich domain (ADAM17-C600Y) that had low residual activity, which could be substantially increased when the cytoplasmic domain was deleted [39]. However, Li et al. observed no increase in the activity of wt ADAM17 upon deletion of its cytoplasmic domain, which is consistent with our conclusion that deletion of the cytoplasmic domain of ADAM17 has no detectable effect on the catalytic activity of the wt protein.

Taken together, stimulation of ADAM17 by IL-1β or anisomycin appears to be regulated by a mechanism that relies on activation of p38 MAPK, but does not require phosphorylation of T735 or the presence of the cytoplasmic domain of ADAM17, at least under the conditions used here. It is tempting to speculate that a yet-to-be-identified membrane protein that interacts with ADAM17 is responsible for its rapid posttranslational activation. There is considerable interest in ADAM17 as a therapeutic target because it regulates EGFR signaling and the release of TNFα. Elucidation of the underlying mechanism of its activation could therefore potentially uncover novel targets for treatment of diseases such as cancer or rheumatoid arthritis. Our results suggest that it will be important to explore mechanisms that do not involve phosphorylation of the cytoplasmic domain of ADAM17 in order to understand the regulation of this principal sheddase for EGFR-ligands and TNFα.

## Supporting Information

Figure S1 **Wildtype and mutant ADAM17 constructs rescue IL-1β-stimulated shedding of alkaline phosphatase-tagged TNFα from primary** ***Adam17−/−*** **chondrocytes.** *Adam17−/−* chondrocytes were prepared as described by Gosset et al. [40] with the exception that E18.5 embryos were used instead of 5-day old mice. The *Adam17−/−* chondrocytes were transfected with the wt or mutant forms of ADAM17 and a reporter construct consisting of an alkaline phosphatase tag attached to the cleavage site and transmembrane domain of human TNFα. Stimulation with IL-1β lead to an increase of shedding activity by wt ADAM17, ADAMT735A or T735D or ADAM17ΔC, but not by the catalytically inactive ADAM17EA mutant. Additionally, IL-1β-stimulated shedding by wt ADAM17 and the T735A, T735D and the ΔC mutants could be inhibited by 10 µM of the p38 MAPK inhibitor SB203580 (SB203), but not by the carrier DMSO. These results represent the average of at least 3 experiments +/− sem. Asterisks indicate significant increase upon addition of a stimulus.
(TIF)
