# Supplementary figures and images for: Interleukin-1 Stimulates ADAM17 through a Mechanism Independent of its Cytoplasmic Domain or Phosphorylation at Threonine 735
Source: PLoS One. 2012 Feb 27;7(2):e31600. doi: 10.1371/journal.pone.0031600 (PMC3288042; doi:10.1371/journal.pone.0031600)

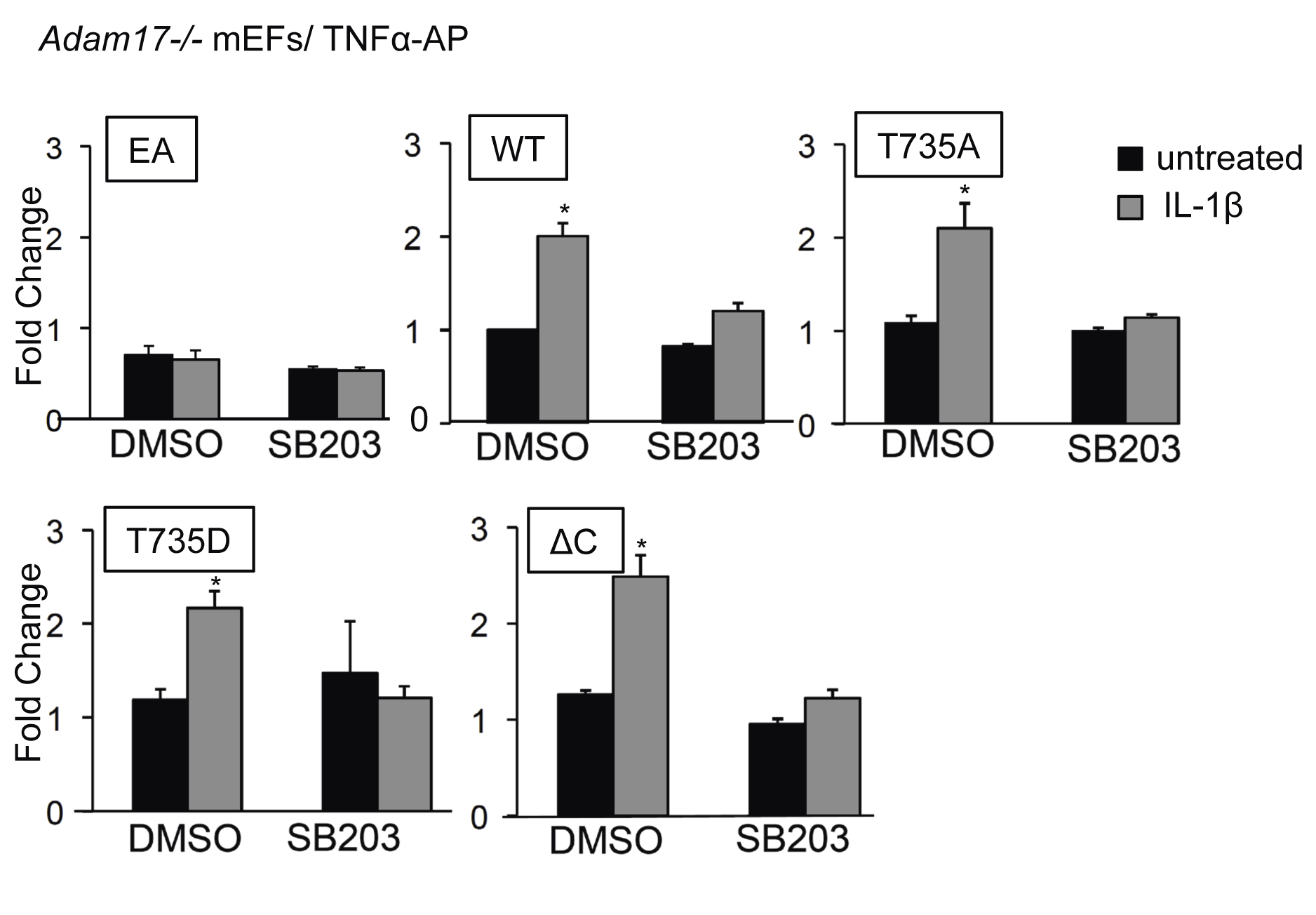

Supplement: Figure S1 — Wildtype and mutant ADAM17 constructs rescue IL-1β-stimulated shedding of alkaline phosphatase-tagged TNFα from primary Adam17−/− chondrocytes. Adam17−/− chondrocytes were prepared as described by Gosset et al. [40] with the exception that E18.5 embryos were used instead of 5-day old mice. The Adam17−/− chondrocytes were transfected with the wt or mutant forms of ADAM17 and a reporter construct consisting of an alkaline phosphatase tag attached to the cleavage site and transmembrane domain of human TNFα. Stimulation with IL-1β lead to an increase of shedding activity by wt ADAM17, ADAMT735A or T735D or ADAM17ΔC, but not by the catalytically inactive ADAM17EA mutant. Additionally, IL-1β-stimulated shedding by wt ADAM17 and the T735A, T735D and the ΔC mutants could be inhibited by 10 µM of the p38 MAPK inhibitor SB203580 (SB203), but not by the carrier DMSO. These results represent the average of at least 3 experiments +/− sem. Asterisks indicate significant increase upon addition of a stimulus. (TIF) [file pone.0031600.s001.tif]
